# Understanding Seizures and Prognosis of the Extreme Delta Brush Pattern in Anti-N-Methyl-D-Aspartate (NMDA) Receptor Encephalitis: A Systematic Review

**DOI:** 10.7759/cureus.18154

**Published:** 2021-09-21

**Authors:** Jashank Parwani, Juan Fernando Ortiz, Ammar Alli, Ayushi Lalwani, Samir Ruxmohan, Hyder Tamton, Victor D Cuenca, Dina Gonzalez, Fatima Anwer, Ahmed Eissa-Garcés, Ivan Mateo Alzamora, Maria Paez

**Affiliations:** 1 Neurology, Lokmanya Tilak Municipal Medical College, Mumbai, IND; 2 Neurology, Universidad San Francisco de Quito, Quito, ECU; 3 Neurology, Larkin Community Hospital, Miami, USA; 4 Medicine, Tishreen University Faculty of Medicine, Lattakia, SYR; 5 Internal Medicine, Universitat de Barcelona, Barcelona, ESP; 6 Internal Medicine, KJ Somaiya Medical College, Mumbai, IND; 7 Medicine, Universidad San Francisco de Quito, Quito, ECU; 8 Medicine, Universidad del Sinu, Cordova, COL; 9 Neurology, California Institute of Behavioral Neurosciences & Psychology, Fairfield, USA; 10 General Medicine, Pontificia Universidad Catolica del Ecuador, Quito, ECU

**Keywords:** extreme delta brush, anmdare, nmda, encephalitis, movement disorder, epilepsy, partial epilepsy

## Abstract

Anti-N-methyl-d-aspartate (NMDA) receptor encephalitis (ANMDARE) is an autoimmune disorder with neurological and psychiatric features. The disease presents with a viral prodrome, followed by psychiatric manifestations. In the next phase, movement disorders or/and seizures occur. Finally, in the last phase, there is a decrease in the level of consciousness. Central hypoventilation and autonomic dysfunction can occur. Recently a unique EEG (electroencephalogram) pattern has been associated with anti-NMDA receptor encephalitis, the extreme delta brush (EDB). Although the association of the EDB with ANMDARE is known by the medical community, its significance is mainly unknown. A systematic review on NMDARE is also scarce. We decided to conduct a systematic review on this topic to consolidate the knowledge and establish the importance of the EDB as a prognostic factor. To conduct this systematic review, we used only studies conducted in humans, written in English, and published in the last 20 years. We used PubMed as a database and searched the following search terms: ("NMDA encephalitis"[Title/Abstract] AND "Epilepsy"[Title/Abstract]) OR (NMDA encephalitis"[Title/Abstract] AND "seizures" [Title/Abstract]) OR ("NMDA encephalitis"[Title/Abstract] AND "extreme delta brush"[Title/Abstract]). The protocol used for this systematic review was the Meta-analyses Of Observational Studies in Epidemiology (MOOSE) protocol, and to analyze the bias of the studies, we used the ROBINS-1 tool.

Eight studies were collected from our search strategy. Our data pulling showed that seizures were present in 178/249 (71.48%) patients. Status Epilepticus was reported in 29/96 (30.20%), and the EBD was seen in 30.89% (55/178) patients with seizures. The range of EDB was 5.9%-33% among the studies. Because the sample size was small, the statistical power was decreased. We had a low overall risk of bias. The wide range in the results could be related to the timing of the EEG recording. EDB was associated overall with increased length of hospital stay, increased ICU admission, and incidence of status epilepticus. The etiology of the EDB remains mainly unknown. However, it has been postulated that in NMDAR encephalitis, there is a disruption of the rhythmic neuronal activity. When antibodies block/target the NMDAR, the rhythmic neuronal activity is disrupted, leading to the unique EDB pattern. Another theory suggests that delta activity is caused because of focal abnormalities in the brain, and the superimposition of the beta waves is related to the alterations of the NMDA receptors.

## Introduction and background

Anti-N-methyl-d-aspartate (Anti-NMDA) encephalitis is an autoimmune disorder with neurological and psychiatric features [1]. The incidence of the disease is 0.15 per 100,000 persons per year [1]. The condition affects both sexes, with a 4:1 ratio in favor of females, and it has a bimodal age distribution, with children (as young as eight months) and adults (20 to 40 years) being the most affected. [1]. Although patients tend to have a longer hospital stay, ICU admission, and hospital costs than in other encephalitides, they are more likely to have a full recovery or recovery with minor sequelae (75%) than severe CNS deficits or death (25%) [2,3]. Mortality from ANMDARE ranges from 2.7% to 11.45% [4]. 

The NMDA receptor is a glutamate-gated ion channel that structurally contains NR1 and NR2 subunits and plays a critical role in synaptic plasticity and memory formation, primarily in frontotemporal, limbic and hypothalamic areas [5,6]. Antibodies to this receptor have been associated with tumors, mainly with ovarian teratoma (50% of female patients older than 18 years) and testicular germ cell tumors (male) [2]. About 20-27% of patients with herpes simplex encephalitis (HSE) develop anti-NMDA antibodies after 2-16 weeks, a phenomenon known as “post-HSE neurological relapsing symptoms” and is related to CNS infections [7]. Cases of anti-NMDA encephalitis have now been reported in patients with recent or current COVID-19 infection [8]. Possible triggers (e.g., tumors or infections) may explain the pathogenesis of the disease [2]. 

There are several phases in ANMDARE. It begins with a prodromal phase characterized by headache, fever, or a week-long viral-like process in 70% of patients [1,5]. The illness phase is characterized by psychiatric symptoms such as psychosis, agitation, hallucinations, mania, and seizures (the latter can appear at any time during the disease) [1,5]. If the receptor antagonism persists, a third phase develops (weeks-months) which consists of orofacial dyskinesias, choreoathetosis, dystonia, rigidity, catatonia, and mutism [1,5]. Finally (months-years) it may present with executive function deficits, memory deficits, and decreased level of consciousness. Although psychiatric symptoms predominate in adults and older adolescents, in children, movement disorders and neurological symptoms are more common. [1,5].

The EDB is a pattern usually seen in premature infants. Besides being found in infants, it seems to be unique and specific in anti-NMDAR encephalitis [9]. The pattern has synchronous and symmetric 1-3 Hz waveforms of 1-3 Hz with a superimposed burst of rhythmic 12-30-Hz activity, which is present continuously on the EEG recording [9]. The pattern is usually seen in frontotemporal regions. This pattern usually disappears after treatment with anti-epileptics, but in some cases may persist for several months [10].

While the association of the EDB with ANMDARE is known by the medical community, its significance is mainly unknown. A systematic review on NMDAR is also scarce. We decided to conduct a systematic review on this topic to consolidate the knowledge and establish the importance of the EDB as a prognostic factor.

## Review

Methods

Protocol

We carried out a systematic review using Meta-analyses Of Observational Studies in Epidemiology (MOOSE) protocol [11].

Eligibility Criteria and Study Selection:

For the Systematic review, only observational studies were included. The studies had to be conducted on humans, written in English, published in the last 20 years. After screening the studies, we only included papers with one of the following characteristics:

(1) Population: Patients diagnosed with NMDAR encephalitis

(2) Intervention: none. 

(3) Comparator: Presence or not of extreme delta brush. 

(4) Outcomes: Functional outcome, mortality. 

Database and Search Strategy:

We used PubMed as a database for the systematic review. The search was conducted between July 1 2021 and September 1 2021. We used an advanced search strategy with the following terms: ("nmda encephalitis"[Title/Abstract] AND "Epilepsy"[Title/Abstract]) OR ("nmda encephalitis"[Title/Abstract] AND "seizures"[Title/Abstract]) OR ("nmda encephalitis"[Title/Abstract] AND "extreme delta brush"[Title/Abstract]) 

Data Extraction and Analysis:

We collected the following information from each paper: 1) Autor(s); 2) year of publication ; 3) Patients characteristics); 4) Use of antiepileptics; 5) EEG changes, including the EDB; 6) Prognosis and scoring.

Bias Assessment:

We used the ROBINS-1 tool for bias analysis of this systematic review [12].

Results

**Figure 1:**
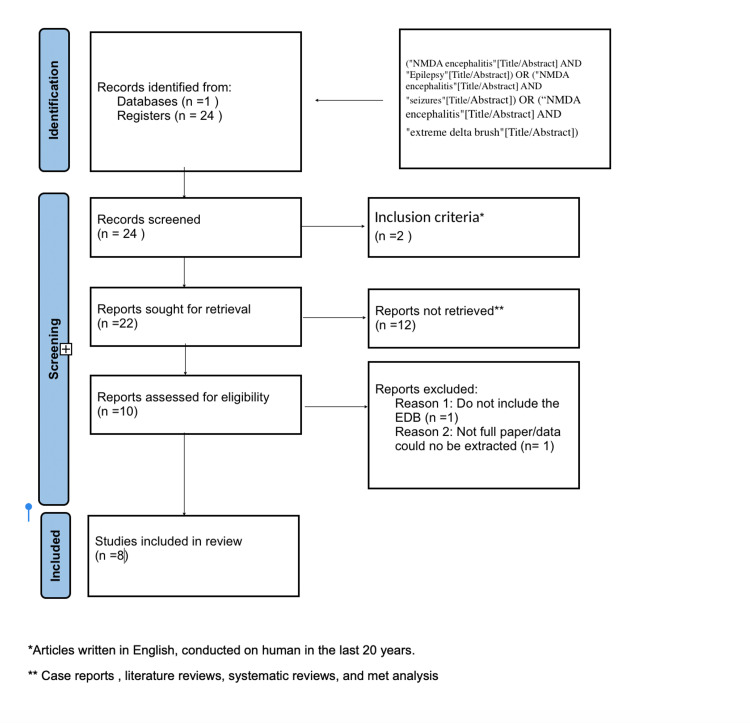
PRISMA Flow chart of the systematic review. PRISMA: Preferred Reporting Items for Systematic Reviews and Meta-Analysis.

Figure 1 shows the results of the study using a Preferred Reporting Items for Systematic Reviews and Meta-Analysis (PRISMA) Flow chart.

Study Characteristics

We found six observational studies discussing the extreme delta brush and seizures on NMDA encephalitis. Table 1 discusses the main findings in these studies [4,10,13,14,15,16].

**Table 1:** Study characteristics of the systematic review. EDB: extreme delta pattern: NMDA: N-methyl-d-aspartate.

Study	Study type	Patient Characteristics	Use of antiepileptics (AED)	EEG changes
Veciana [10] (2015)	Observational Study	Total- 15 patients were included 11 females (73%) 4 males (27%) Mean age- 37.4 years Seizures occurred in 9/15 (60%) and Status epilepticus in 5/9 (55.5%).	9 patients (60%) received various antiepileptic drugs (AEDs), and 5/15 (33%) patients received sedative drugs, 2 of which were used to induce pharmacologic coma.	Diffuse background slowing was seen in 7 (46%). Generalized delta activity was seen in 7 (46%), focal delta activity in 7 (46%), and increased beta activity in 8 (53%) patients. EDB was observed in 5 patients (33%).
Espinola-Nadurille [13] (2019)	Observational study	58 patients with a definite diagnosis of ANMDARE were included Mean age- 25.9 years 30 (51%) females28 (49%) males 45 (77%) had seizures and 15 (33.3%) of those had Status epilepticus.	Not described	EEG was abnormal in 52 patients (89%). Delta brush activity was seen in 11 (19%) patients.
Schmitt [14] (2012)	Case series	23 patients with a definite diagnosis were included Median age- 24 (range 18-55) years. 19/23 patients (83%) were female. Clinical seizures before or during hospitalization occurred in 18 patients (78%), the median length of hospitalization was 44 (range 2–200) days.	Phenobarbital, benzodiazepines	Normal EEG- 8.7% Electrographic seizures- 60.1% Clinical seizures- 60.9% EDB- 30.4%
Aungsumart [4] (2018)	Observational study	31 patients The median age 19 years (IQR 15.0–31.0) Females-19 (61.8%) Males- 12 (38.7%) Seizures- 16 patients (51.1%)	Not described	EDB was found in 8 out of 30 (26.7%) patients with abnormal EEG.
Wang, et al. [15] (2019)	Prospective cohort study	16 patients with anti-NMDAR encephalitis 15 patients served as a control group Mean age- 29.5 years 11 females 5 males 87.5% (14/16) had seizures at some point.	Only 2 patients were on AEDs at the study time. Topiramate and Oxcarbazepine were used.	EDB was present in 5/16 (31.25%) patients.
Zhang et al. [16] (2019)	Retrospective cohort study	34 patients with anti-NMDAR encephalitis; Median age: 20 months; Sex: Males (16) Females (18) ; Seizures: 18 cases(52.9%) ; Single seizures- 2/18 (11.1%) ; Repetitive seizures- 16/18 (88.8%) Status epilepticus.- 9/18 (50%) ; Generalized seizures: 5/18 (27.7%) Focal seizures: 11/18 (61.1%) ; Mixed seizures: 11%	14 out of 18 patients (77.7%) with seizures accepted AEDs and seizure freedom was achieved in 12 out of 14 (85.7%) patients at the last follow-up. Ten of these 12 (83.3%) patients withdrew from AED treatment within 1 year.	EDB patterns were recorded in 2/34 (5.9%) patients and disappeared 6 months after immunotherapy
Jeanin-Mayer (2019)	Observational study	24 patients with confirmed Anti-NMDA Receptor Encephalitis were enrolled. Mean age- 20.7 years Females- 21 (87%) Males- 3 (13%) Seizures- 18/24 (75%) No Status Epilepticus was recorded.	Antiepileptic drugs were administered to all the patients during the course of their disease, however, benzodiazepine could be given for behavioral causes as well. AEDs included benzodiazepines, barbiturates, lacosamide, levetiracetam, lamotrigine, etc.	All patients underwent several EEGs. The median number of EEGs per patient was 8. Slow waves were recorded in all patients. Spikes were present in 15 (62%), Excessive Beta Activity (EBA) in 17 (71%), and Generalized Rhythmic Delta Activity (GRDA) in 12 (50%) patients. EDB was present in 14 (58%) patients. EBA appears first, followed by EDB and then GRDA with a respective median time of appearance of 10, 16.5, and 21.5 days.
Viswanathan (2020)	Retrospective cohort study	48 patients who were diagnosed to have NMDARE were enrolled. Females- 39 (81%) Males- 9 (19%) Mean age- 14.5 years. Seizures were present in 40 (82%) patients.	Not described	The most common EEG pattern that was noted was diffuse slowing (n= 20) followed by generalized rhythmic delta activity (n= 9), focal spikes, and slowing (n= 8 each). EDB was seen in only 3 EEGs.

Table 2 shows the outcome and prognosis of the EDP [4,10,13,14,15,16].

**Table 2:** Outcomes and prognosis of the EDP in the studies of the systematic review. EDP: extreme delta pattern: NMDA: N-methyl-d-aspartate, mRS: modified Rankin scale; EBA: excessive beta activity; GRDA: generalized rhythmic delta activity.

Study	Outcomes and prognosis of the EDP
Veciana et al [10] (2015)	No differences in age and sex were observed when comparing patients with and without EDB, however, all the men in the series (4/15) had an EEG without EDB. All patients with EDB had seizures vs only 4 (40%) patients without EDB. Moreover, all patients with EDB suffered recurrent seizures and were diagnosed with status epilepticus, and none of the patients without an EDB pattern suffered from status epilepticus. MRI abnormalities were more common in patients without EDB than those with EDB. No differences were observed in evolution after 6 months. No patients had reported non provoked seizures in the follow-up, but 1 patient (6%) had a persistent abnormal EEG with focal epileptiform activity.
Espinola-Nadurille et al. [13] (2019)	Status epilepticus was more frequent in patients without catatonia. Mortality was present in 10% of the total sample. It was associated with status epilepticus and was less frequent in the catatonia group.
Schmitt et al. [14] (2012)	None of the patients with EDB responded clinically or electrographically, despite the use of benzodiazepines and other IV antiepileptic drugs. Patients with EDB were associated with a trend toward worse outcomes at discharge, with a mean modified Rankin Scale score of 4.0 +/- 0.8 compared with 3.1+/- 1.1 in patients without EDB. Patients with EDB had more protracted hospital courses, with a median of 126 (range 70-200) days in the hospital and 14 (5-123) days undergoing cEEG monitoring compared with 36 (2142) hospital days and 3.5 (120) cEEG days in patients without the pattern. Patients without EDB were more likely to have an abnormal MRI than those with EDB.
Aungsumart et al. [4] (2018)	EEG patterns were not related to worse prognosis, but the study failed to differentiate if there between patients with EDB and without EDB.
Wang et al. [15] (2019)	2/7 patients on first-line therapy had seizures. Of the 9 patients who received second-line drugs, none had seizures. Seizures and EDB did not significantly differ in the first line and second line groups, However, seizures were more frequent in patients who received only first-line treatment.
Zhang et al. [16] (2019)	Only 2 (11.1%) patients reported seizures at the last follow-up. The median duration of follow-up was 20 months The median mRS score was 5 before immunotherapy, and it decreased to 0 after 3–6 months of initial immunotherapy. At the last follow-up, 29 patients (85.2%) had fully recovered, 1 patient (2.9%) exhibited mild deficits. 4 patients (11.8%) exhibited severe deficits, 2 with intractable epilepsy. There were no deaths. They concluded that seizures, psychiatric manifestations, and cognitive dysfunction are the most common symptoms of pediatric NMDA encephalitis. While the presence of EDB should prompt suspicion of NMDA encephalitis, other possible etiologies should not be ignored.
Jeanin-Mayer (2019) [17]	The EEG patterns occurred in a definite chronological organization, with EBA appearing first, followed by EDB and then GRDA. None of these patterns was associated with seizures, but GRDA was associated with abnormal movements. In the study, EDB was not associated with epileptic seizures or status epilepticus, as reported by Veciana et al. (2015), and was not correlated with bad outcomes.
Viswanathan (2020) [18]	The mRS scores were recorded at first admission, discharge, and at last follow-up. On admission, 33 patients had mRS scores of 4-5. At last follow-up (24 months), 37 patients had mRS scores < 2. Both patients who had EDB in our study presented with alteration in consciousness and thereafter improved with treatment. After six months of treatment, both were asymptomatic. The prognostic value of EDB is not clear.

Extreme Delta Brush

**Figure 2:**
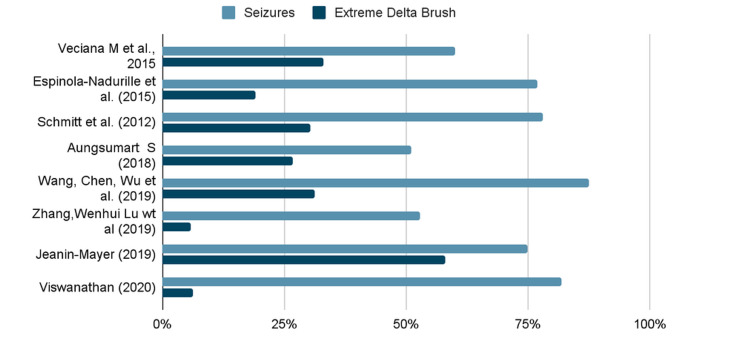
Prevalence of EDP. EDP: extreme delta pattern.

In Figure 2 we see the prevalence of seizure and delta brush in each of the studies. 

Our pooled data show that the prevalence of seizures among the 8 studies was 178/249 (71.48%) patients. The prevalence of EDB pattern was 22.08% (55/249) in all patients and 30.89% (55/178) in patients with seizures.

Table 3 shows the bias analysis of the systematic review using the Risk Of Bias In Non-randomised Studies - of Interventions (ROBINS-1) tool [4,10,13,14,15,16].

**Table 3:** Bias analysis using the ROBINS-1 tool. ROBINS-1: Risk Of Bias In Non-randomised Studies - of Interventions.

	Confounding	Selection bias	Classification of intervention	Deviation from intervention	Missing data	Measurement of the outcome	Selection of reported result
Veciana et al. 2015 [4]	Low risk	Moderate risk	Low risk	Moderate risk	Moderate risk	Low risk	Low risk
Espinola-Nadrulle et al. 2015 [10]	Low risk	Low risk	Low risk	Moderate risk	Low Risk	Low risk	Low risk
Schmit et al. 2012 [13]	Low risk	Low risk	Moderate risk	Moderate risk	Moderate risk	Moderate risk	Low risk
Aungsumart et al. 2018 [14]	Low risk	Moderate risk	Low risk	Low risk	Low risk	Low risk	Low Risk
Wang et al [15]	Low risk	Low risk	Los risk	Low risk	Low risk	Low risk	Low Risk
Zhang et al. 2019 [16]	Low risk	Low risk	Low risk	Low risk	Low risk	Low risk	Low Risk
Jeanin-Mayer (2019) [17]	Low risk	Low risk	Moderate risk	Moderate risk	Moderate risk	Moderate risk	Low risk
Viswanathan (2020) [18]	Low risk	Low risk	Low Risk	Low risk	Low risk	Low risk	Low risk

Additionally, the authors mentioned other limitations: According to Aungsumart et al., their study was limited mainly due to a retrospective study design [14].

Limitations in Wang et al.’s study were due to sample size and study design that can lead to selection bias. Additionally, this study did not focus on mild cases of anti-NMDAR encephalitis and had difficulty following up with patients [15].

Zhang et al.’s study was limited due to selecting the sample only at Children’s Hospital of Fudan University in Shanghai, including diagnosis and treatment. Besides, it has the characteristics of a retrospective study [16].

According to Viswanathan et al., this study was limited mainly because the study design is retrospective and possibly due to no use of cEEG; they did not report enough data about the abnormalities [17].

Limitations in Jeannin-Mayer et al.'s study were a small median of cerebral MRI performed in every patient and the lack of monitoring each patient using the EEG to identify the appearance abnormalities [18].

Discussion

Seizures are a common clinical manifestation in ANMDARE along with psychiatric manifestations and movement disorders. According to a systematic review by Gillinder et al, seizures are present in 294/446 (65.9%) cases of NMDA encephalitis patients [19]. Our pooled data show that seizures were present in 178/249 (71.48%) patients. Status Epilepticus was reported in 29/96 (30.20%) patients that suffered from seizures in our studies.

The types of seizures are variable and patients may have generalized, partial, or mixed type seizures [16]. In the study by Zhang et al., who compared seizures in ANMDARE patients with and without teratomas, complex partial seizures were more common in the teratoma group [20]. There are no systematic reviews that include pooled data of EDB. In our study, it was present in 30.89% (55/178) patients with seizures.

Etiology

The etiology of the EDB pattern remains mainly unknown. Additionally, defining the pattern of ictal vs interictal is also not known. The EDB pattern is a mix of beta waves with superimposed delta activity. The beta activity tends to occur in a burst, synchronized, and diffuse fashion, while the delta activity tends to be more localized frontally [14]. Schmit et al proposed that there is a disruption of the rhythmic neuronal activity in ANMDAR encephalitis. When antibodies block/target NMDAR, the rhythmic neuronal activity is disrupted which could lead to the unique EDB pattern [20].

The term EDB is derived from its resemblance to the delta brush pattern seen in premature infants, also known as beta-delta complexes [20]. The typical neonatal delta brushes are a combination of delta frequency transients with superimposed 8−20 Hz fast activity. However, there are differences between the two. Neonatal delta brushes are usually symmetric but not synchronous and are seen less commonly in the frontal regions of the head. In contrast, the EDB pattern is often symmetric and synchronous and is typically seen broadly across all head regions [20]. Another theory proposed that delta activity is caused by focal abnormalities in the brain, and the superimposition of the beta waves is related to the alterations of the NMDA receptors [21]. 

Jeanin-Mayer et al. explained additional EEG patterns, excess beta activity (EBA), and generalized rhythmic delta activity (GRDA). EBA is a pattern ranged 14-20 hertz that is also found in benzodiazepine and barbiturate use, while GRDA is present in several comatose patients. All three patterns were found frequently in ANMDARE patients. A clear chronological organization in the course of the disease was evidenced. They found that EBA appears first, followed by EDB and then GRDA with a respective median time of appearance of 10, 16.5, and 21.5 days [17].

Outcomes

In all our studies reviewed, the combined prevalence of the EDB pattern was 22.08% (55/249) in all patients and 30.89% (55/178) in patients with seizures. Overall, two studies concluded worse short-term outcomes in patients with EDB but the other two studies displayed a lack of such evidence.

In the Veciana et al study, patients who suffered status epilepticus and had EDB on EEG tended to have fewer abnormal findings on MRI than patients without EDB. Additionally, patients with EDB patterns did not have a worse long-term outcome, but they needed more aggressive treatment with anesthetic coma and spent more days either in the hospital or the intensive care unit setting, thus confirming worse short-term outcomes [10]. According to Schmit et al, patients with EDB were hospitalized and monitored with cEEG longer and demonstrated a trend toward a worse outcome. These findings suggest that the EDB pattern may be a marker of more severe disease and perhaps worse outcomes [14]. On the other hand, Aungsumart et al concluded that abnormal EEG patterns were not associated with poorer outcomes. However, this study did make a direct comparison of patients with and without EDB and clinical outcomes [4]. Similarly, Jeanin-Mayer and Viswanath stated that the EDB pattern was not related to more seizures or status epilepticus, and did not correlate with worse outcomes [17].

Both Schmit and Veciana supported the notion that patients who had EDB tended to have fewer abnormal findings on MRI than patients without EDB [10,14].

In terms of seizure-related outcomes, Alvarez et al. suggested that the need for treatment of seizures and movement disorders was predictive of unfavorable early outcomes. However, this was not confirmed by other studies [21]. Viswanathan LG et al. said that there was no significant difference in outcomes such as seizure recurrence, modified Rankin score (mRS) at follow-up/discharge, or relapse between groups of patients who had EEG abnormalities in the first EEG and with those who did not. According to Zhang et al., the prevalence of seizures was higher in patients under 6 years of age. The most common type was focal seizures and patients with ANMDARE tended to have repetitive seizures as compared to single seizures or status epilepticus [16]. Most patients achieved seizure freedom, so long-term use of anti-epileptic drugs may not be necessary for the pediatric population. According to the literature, adults tend to present with focal seizures and children tend to present with generalized seizures. In the Zhang study, the EDB was only seen in 11.1% of patients, which is lower than the other studies. The author suggested that the difference could be related to the timing of the EEG recording [16].

From the other EEG patterns studied by Veciana (EBA and GRDA), neither was associated with seizures, but GRDA was associated with abnormal movements [17].

In the Spianola et al study, neither seizures nor the EDB was related to catatonia. However, patients with Catatonia had more status epilepticus [13].

Response to treatment

Zhang Y et al. studied the efficacy of a newer modality, Therapeutic Plasma Exchange (TPE) in patients with severe ANMDARE (mRS score of 4-5). They found that patients in the TPE group showed greater clinical improvement at 1 and 2 months after treatment, compared to the non-TPE group. There was no significant improvement at 3, 6, and 12-month follow-ups. It also helped to decrease the dose of required AEDs [22].

In the study of Wang et al., there was a statistically significant difference between patients who receive only first-line treatments (steroids, intravenous immunoglobulin, plasmapheresis) vs first-line plus second-line therapy (rituximab, cyclophosphamide) in the relationship of seizures or EDB. Overall, using second-line therapy significantly improved verbal episodic memory outcomes in patients with anti-NMDAR encephalitis [15].

A variety of anti-epileptics have been used for the management of seizures in ANMDARE. These include leviteracetam, lacosamide, phenytoin, oxcarbazepine and topiramate. In certain cases, patients required pharmacologic coma with phenobarbital [10]. Patients usually (85%) attain freedom from seizures and do not need AEDs after 1 year [16]. In the study by Zhang, seizures persisted in only 2/34 (11.1%) patients at the last follow-up at 20 months [16]. This pattern may be found when the patients first present to the hospital and usually disappears after treatment with anti-epileptics, but in some cases, it may persist for several months [14].

## Conclusions

The etiology of the EDB remains essentially unknown. However, it has been postulated that in ANMDAR encephalitis, there is a disruption of the rhythmic neuronal activity. When antibodies block/target NMDAR, the rhythmic neuronal activity is disrupted, leading to the unique EDB pattern. Another theory suggests that delta activity is caused because of focal abnormalities in the brain, and the superimposition of the beta waves is related to the alterations of the NMDA receptors.

There was wide variability in the prevalence of EDB (6%-58%) in our sample which could be related to the timing of the EEG recording. The pooled prevalence was 30.89% (55/178) in patients with seizures. Overall, two studies concluded worse short-term outcomes in patients with EDB but the other two studies displayed a lack of such evidence. Some patients with EDB had prolonged hospital stays, increased ICU admission, and a higher frequency of status epilepticus. These findings suggest that the EDB pattern may be a marker of more severe disease and worse short-term outcomes, while long-term outcomes are not affected. Since treatment with TPE in severe anti-NMDAR encephalitis can improve short-term clinical outcomes including epilepsy, we suggest that every patient with EDB pattern may benefit from this treatment.
